# Trehalose Activates CRE-Dependent Transcriptional Signaling in HT22 Mouse Hippocampal Neuronal Cells: A Central Role for PKA Without cAMP Elevation

**DOI:** 10.3389/fnmol.2018.00386

**Published:** 2018-10-22

**Authors:** Erik Maronde

**Affiliations:** Department of Medicine, Institute for Cellular and Molecular Anatomy, Goethe University, Frankfurt, Germany

**Keywords:** trehalose, CRE-luciferase, pCREB, hippocampal neuronal cell line, signaling

## Abstract

Cyclic adenosine 3′,5′monophosphate (cAMP) regulated element binding protein (CREB) is a transcription factor involved in many different signaling processes including memory storage and retrieval. The mouse hippocampal neuronal cell line HT22 is widely used as a model system for neuronal cell death and cellular signal pathway investigations. For the present work a variant of HT22 with a stably expressed CRE-luciferase (CRE-luc) reporter (HT22CRE) is introduced, characterized and used to investigate cAMP-dependent and independent CRE-dependent signal processes. Trehalose (Mykose or 1-α-Glucopyranosyl-1-α-glucopyranosid) is a naturally occurring disaccharide consisting of two α,α′,1,1-glycosidic connected glucose molecules in a wide range of organisms but usually not found in mammals. Trehalose has been shown to activate autophagy, a process which regulates the degradation and recycling of proteins and organelles. The exact processes how trehalose application works on mammalian neuronal cells is not yet understood. The present work shows that trehalose application dose-dependently elevates CRE-luc activity in HT22 cells and acts synergistically with cAMP-elevating agents. In this pathway cAMP-dependent protein kinase (PKA) appears to be the most important factor and the stress kinase p38 and protein tyrosine kinases (PTKs) act as modulators.

## Introduction

### Trehalose, Autophagy and Human Therapy

Trehalose (Mykose or 1-α-Glucopyranosyl-1-α-glucopyranoside) is a naturally occurring disaccharide consisting of two α,α′,1,1-glycosidic connected glucose molecules in a wide range of organisms but usually not produced in mammals. It plays an important role in hibernation of invertebrates and can be used to preserve deep-frozen mammalian cells (Bailey et al., 2015), tissues and organs (Richards et al., 2002; Iturriaga et al., 2009). Trehalose has been shown to activate autophagy, a process which regulates the degradation and recycling of proteins and organelles. Trehalose is well tolerated by human patients. Doses of 30 g/patient/day intravenous (which would result in a transient concentration of approximately 20 mM in blood) and 50 g/day per patient are considered safe (Richards et al., 2002). Such doses of trehalose have been utilized in experimental human studies for the treatment of Oculopharyngeal Muscular Dystrophy (OPMD; Argov, 2015). The exact processes how trehalose application works on mammalian cells, including neuronal cells, is not yet understood, but extra- and intracellular action as well as mTor-dependent and -independent mechanisms have been discussed (Sarkar et al., 2007; Iturriaga et al., 2009; Casarejos et al., 2011; Holler et al., 2016; Martano et al., 2017; Lee et al., 2018).

### CREB Phosphorylation and CRE-Dependent Signaling

Cyclic adenosine 3′,5′monophosphate (cAMP) regulated element binding protein (CREB) binds in its serine 133 phosphorylated and dimerized form (pCREB) to a palindromic DNA sequence (5′-TGACGTCA-3′; CRE; Shaywitz and Greenberg, 1999). Multiple signaling pathways and a number of different protein kinases among which cAMP-dependent protein kinase (PKA) and Calcium/Calmodulin-dependent kinase II/IV (CaMKII/IV) are the most intensely investigated have been described to phosphorylate CREB at serine 133 (Mayr and Montminy, 2001; Kandel, 2012). CREB phosphorylation is involved in synaptic plasticity processes including those necessary for memory formation and neurodegeneration (Benito and Barco, 2010). In a wider approach testing the modulation of signaling processes which involve the phosphorylation of CREB and the activation of CRE-dependent transcriptional pathways we surprisingly observed that application of the disaccharide and “anti-aging drug” trehalose elevated the phosphorylation of CREB at serine 133 (pCREB) as well as CRE-luciferase (CRE-luc) activity. Trehalose also enhanced, as described in other cells types before, phosphorylation of the protein kinase p38 (Burg et al., 2007). The elevation of pCREB and CRE-luc activity by trehalose is also remarkable since phosphorylation of CREB is long known to be involved in neuroprotection after ischemia (Walton et al., 1996; Lee et al., 2005), for memory formation, storage and retrieval in the hippocampus (Kandel, 2012; Rawashdeh et al., 2016, 2018). Moreover, trehalose was recently described to play a role in neuroprotection (Sarkar et al., 2007) although the mechanism remains controversial (Lee et al., 2018).

### Trehalose, Phospho-p38 and CREB

Trehalose has been described to elevate the phosphorylation of the cellular stress-related kinase p38 (Burg et al., 2007) and p38 has also been shown to be able to phosphorylate CREB at serine 133 directly (Tan et al., 1996).

### Trehalose, Forskolin, PKA and CRE-Signaling

Concentrations of trehalose like those used for autophagy induction in the literature were to my knowledge not shown to elevated CRE-luc activity before. It was also not shown before that trehalose acts synergistically in combination with the known adenylate cyclase activator and thereby cAMP-elevating diterpene forskolin (Seamon et al., 1981; Seamon and Daly, 1986) or the well-described PKA-activating phosphodiesterase-resistant cAMP analog Sp-cAMPS (Schwede et al., 2000; Fricke et al., 2004). Surprisingly, after 1–3 h trehalose was inhibitory in respect to the effects of forskolin, but at later times of exposition (6–12 h) synergistically activated the responses to cAMP-elevating agents.

Due to the central role that CRE-dependent signaling plays in many physiological pathways including neuroprotection, as well as memory formation, consolidation and retrieval this novel action of trehalose alone and in combination with cAMP elevating agents appears to be of potential physiological importance at least for hippocampal neuronal cells.

With the advantage of being able to measure CRE-luc activity in a time-resolved manner in living cells the effect of trehalose in HT22CRE cells was found to be distinctively different to that of the other CRE-luc-activating stimuli in both the timing of the maximal response (which is later than that of forskolin) and the interaction of trehalose with forskolin and Sp-cAMPS.

## Materials and Methods

### Materials

Trehalose was from AppliChem (Heidelberg, Germany) and dissolved directly in medium. Forskolin (Seamon et al., 1981; Pinto et al., 2008) was dissolved in dimethylsulphoxide (DMSO) and purchased from Sigma-Aldrich (Darmstadt, Germany). *Rp*-8-Br-cAMPS (Gjertsen et al., 1995; Schwede et al., 2000) and *Sp*-cAMPS (Fricke et al., 2004) were dissolved in water and purchased from BioLog Lifescience Institute (Bremen, Germany). GW856553X, BMS582949 and the cAMP ELISA Kit were from Cayman (Ann Arbor, MI, USA). SB203580 was from LC Laboratories (Woburn, MA, USA). SP600125 was from Tocris (Ellisville, MO, USA), U0126 and LY294002 were from Cell Signaling Technologies (Bad Nauheim, Germany), Genistein and IC261 were from Calbiochem (San Diego, CA, USA). D-Luciferin (potassium salt) was from Promega (Heidelberg, Germany) and dissolved directly in cell culture medium. Reagents or appropriate vehicle were applied to the media for the indicated periods.

### Cell Culture

Immortalized mouse hippocampal cells (200,000 per well in a 6-well plate; kindly supplied by Dr. David Schubert) in DMEM with 10% FBS, penicillin/streptomycin (Pen/Strep; 100 U/ml) and GlutaMax (Invitrogen/Thermo Fisher, Darmstadt, Germany) were transfected using FuGene HD (Roche, Mannheim, Germany) and a commercially available CRE-luc plasmid (pGL4.29[luc2P/CRE/Hygro]) from Promega (Heidelberg, Germany). After leaving the cells with the transfection agents overnight, medium was changed to DMEM with 10% FBS, Pen/Strep (100 U/ml) and GlutaMax supplied with Hygromycin (250 μg/ml, Enzo Lifesciences, Lörrach, Germany) for at least 3 days. Cells were then washed three times with sterile HBSS. Those cells still attached to the substrate were enzymatically detached, counted (Scepter 2.0, Merck-Millipore, Darmstadt, Germany) and supplied with medium containing Hygromycin for selection of stably transfected clones. Clones showing induction of CRE-luc-activity were expanded and frozen at a density of 1.5 million/ml/vial at −80°C in freezing medium (IBIDI, München, Germany). These cells were named HT22CRE. Cells thawed from these preparations were given the passage number 1 and used up to passage 15. Cells were passaged once a week at a density of 100,000 cells per 75 cm^2^ flask. HT22 wt and HT22CRE were equally healthy (Supplementary Figures S3–S6) and morphologically very similar (Supplementary Figures S5,S6). Trehalose (150 mM) did not affect mitochondrial lactate dehydrogenase activity as estimated by the WST-1 assay (Supplementary Figure S2).

### Measurement of Intracellular cAMP

Intracellular cAMP was determined in HT22CRE cells using acid extraction and a commercial ELISA (Cayman, Ann Arbor, MI, USA; Item no. 581001) according to the supplier’s protocol. In brief cells were plated either at 10,000 cells per well on a 96-well plate or 40,000 cells per well on a 24-well plate, left over night and exposed with the indicated substances for the indicated times. At the indicated times (1 h, 6 h and 12 h) medium was removed and 100 μl (96-well plate) or 400 μl (24-well plate) 0.1 M HCl was added. These plates were then either frozen at −20°C for later processing or cells were directly scraped off, transferred into a fresh tube and centrifuged. Fifty microliter of this extract supernatant were measured in the cAMP ELISA.

### Western Blot

Forty-thousand cells per well in a 24-well plate were plated out in 500 μl/well, left overnight in DMEM with 10% fetal calf serum (FCS), Pen/Strep and GlutaMax (Invitrogen/Thermo Fisher, Darmstadt, Germany). At the next day, medium was changed to DMEM and supplements without FCS. The treatments were applied the following day. At the end of each experiment, medium was removed and cells were immediately lysed in double concentrated (2×) lithium dodecylsulfate sample buffer (LDS; Invitrogen/Thermo Fisher, Darmstadt, Germany). Cell extracts were sonified five times for 3 s using a 24-kHz ultrasound sonifier (Dr. Hielscher, Teltow, Germany), heated for 10 min at 70°C, chilled on ice and centrifuged for 5 min at 13,000 rpm in an Eppendorf cap centrifuge (Eppendorf, Hamburg, Germany). Samples were either used immediately for gel electrophoretic separation on Bis/Tris gels (NuPage 4%–12% gradient gels, MES buffer, Invitrogen/Thermo Fisher, Darmstadt, Germany) or stored at −20°C. After the run gels were blotted using iBlot and PVDF membrane (Invitrogen/Thermo Fisher, Darmstadt, Germany) washed with Tris-buffered saline (pH 7.6) containing 0.1% (vol/vol) Tween-20 (TBS-T; Sigma-Aldrich, Darmstadt, Germany), blocked for 1 h at room temperature using Rotiblock (Roth, Karlsruhe, Germany) and incubated with primary antibodies diluted in Rotiblock overnight at 4°C (Wicht et al., 1999; Benz et al., 2010; Maronde et al., 2011). The antibodies used were mouse anti-ß-actin (1:10,000; Sigma-Aldrich, Darmstadt, Germany), mouse anti GAPDH (1:5,000; Origene, Rockville, MD, USA); rabbit anti-phospho-CREB (87G3; 1:1,000) and anti-phospho-p38 (pp38; 1:1,000; all from Cell Signaling Technologies, Bad Nauheim, Germany). After incubation with the first antibody, membranes were washed three times for 2 min with TBST and incubated with the appropriate secondary HRP-coupled antibodies against rabbit (sc2054; 1:50,000; Santa Cruz Biotechnology, Heidelberg, Germany) or mouse (1:50,000; DAKO, Hamburg, Germany) in Rotiblock for 1 h at room temperature. Membranes were washed with TBS-T five times for 2 min. Signal detection was performed using the chemiluminescent substrates Luminata forte (Merck-Millipore, Darmstadt, Germany), AceGlo (PeqLab, München, Germany) or GE Select (GE Healthcare, Darmstadt, Germany). For recording the chemiluminescence images a CCD camera- equipped luminescence analysis system (Quantity One, ChemiDoc XRS, Bio-Rad, Hercules, CA, USA) as described (Benz et al., 2010; Maronde et al., 2011) or a Vilber Fusion XL system (PeqLab, München, Germany) was used. A detailed description of the (semi) quantification procedure is provided in the supplements (Supplementary Figure S9).

### Determination of Luminescence Activity (RLU)

If not indicated else 10,000 HT22CRE cells per well of a 96-well plate was plated out in 100 μl volume and left in the incubator at 37°C, 5% CO_2_ and 100% humidity overnight for attachment. Experiments were performed in 200 μl medium per well containing 250 μM Luciferin (if not indicated else) and measured in a luminometer (BMG Lumistar or Berthold Centro LB960) at 35°C for 0.1 s per well. Every experiment was executed on both luminescence readers and repeated at least twice. Luminescence data are displayed as relative luminescence units (RLU). Cells were measured after different times of incubation (e.g., every 15 min) with and without the indicated chemical agents.

### Statistical Analysis

Signal intensities of the digitized images were analyzed using a combination of densitometry and volumetry as described in detail before (Wicht et al., 1999) either using the inbuild software of the luminescence readers or using the program QuantiScan by Biosoft (Oxford, United Kingdom). Each area/density value for a specific protein band was normalized against the corresponding ß-actin or GAPDH signal of each extract. A detailed description of the (semi)quantification procedure is provided in the supplements (Supplementary Figure S9).

Two group comparisons with groups larger than two were performed using one-way ANOVA, followed by the Bonferroni *post hoc* test. The criterion of significance was *P* < 0.05, with analysis performed using GraphPad Prism 5.1d (GraphPad, San Diego, CA, USA).

## Results

**Figure 1:**
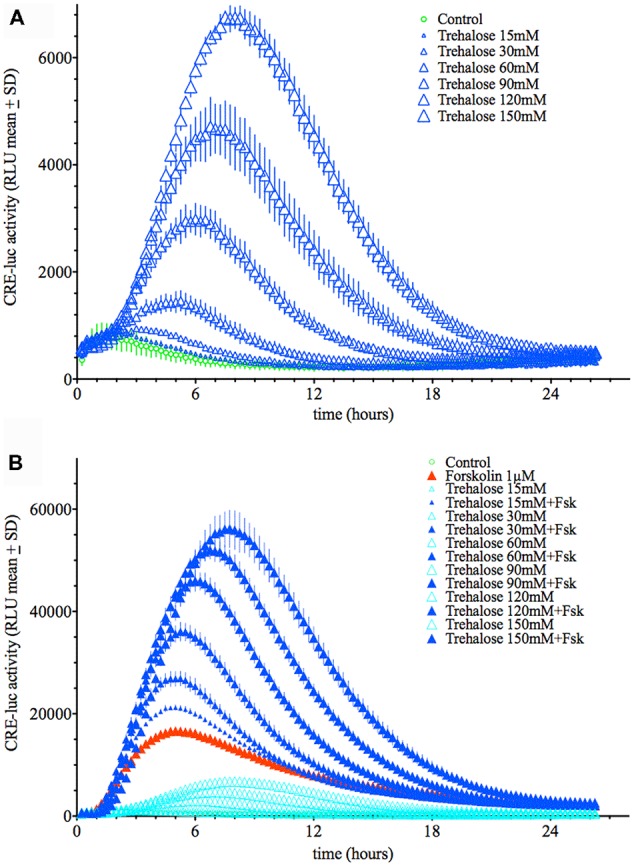
**(A)** Dose-dependent elevation of Cyclic adenosine 3′,5′ monophosphate (cAMP) regulated element-luciferase (CRE-luc) activity by trehalose. The higher the dose of trehalose, the higher and later is the maximal activity (approximately 8 h at 150 mM). Note that the threshold dose is 30 mM. **(B)** Dose-dependent elevation of CRE-luc activity by increasing doses of trehalose with forskolin (1 μM). Note that beginning at 15 mM trehalose, which itself has no significant effect, every dose of trehalose synergistically enhances the effect of forskolin. Shown are the mean relative luminescence units (RLU) values with standard deviation (SD) of *N* = 4 for each time point.

Application of trehalose dose-dependently elevated CRE-luc activity in HT22 cells with a threshold concentration of 30 mM (Figure 1A; Supplementary Figures S4, S10–S12). The time from application to the point of maximal CRE-luc activity was significantly longer for trehalose (approximately 9 h) than for forskolin (approximately 5 h; Figure 1B).

**Figure 2:**
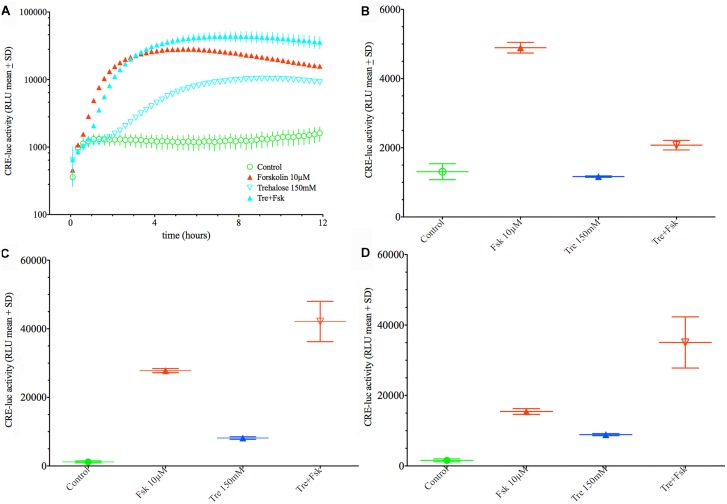
Comparison of the effect of 150 mM trehalose on forskolin-mediated CRE-luc activity at 1 h, 6 h and 12 h of stimulation. The graph on the upper left side **(A)** shows CRE-luc activity of untreated cells (Co), forskolin-treated cells (Fsk 10 μM), trehalose (Tre 150 mM) and the combination of the latter (Tre+Fsk) during the first 12 h of the treatment measured every 15 min starting at 15 min after application of the agents and the vehicle. The bar graph at the upper right side **(B)** shows that after 1 h of stimulation forskolin (10 μM) significantly elevated CRE-luc activity (*p* < 0.0001), whereas trehalose is not significantly elevated compared to control levels. The combination of forskolin with trehalose is significantly lower compared to forskolin (*p* < 0.0001) so that at this time trehalose inhibits forskolin-mediated CRE-luc activity. **(C)** At 6 h of stimulation both forskolin (*p* < 0.0001) and trehalose (*p* < 0.05) significantly elevated CRE-luc activity. The combination of forskolin with trehalose is significantly higher than that of forskolin (*p* < 0.001) or trehalose (*p* < 0.0001) alone and higher than the (theoretical) addition of the forskolin and the trehalose effect. **(D)** At 12 h of stimulation both forskolin (*p* < 0.0001) and trehalose (*p* < 0.05) significantly elevated CRE-luc activity. The combination of forskolin with trehalose is significantly higher than that of forskolin (*p* < 0.0001) or trehalose (*p* < 0.0001) alone and higher than the (theoretical) addition of the forskolin and the trehalose effect.

When applied in combination trehalose and forskolin showed an unexpected bi-phasic behavior. During the first hours of application trehalose (150 mM) alone did not elevate CRE-luc activity over the control levels (Figures 2A,B). Trehalose applied in combination with forskolin significantly inhibited CRE-luc activity compared to forskolin application (Figures 2A,B). After 6 h forskolin application still significantly elevated CRE-luc activity, as did trehalose alone, albeit less strongly. However, the combination of both trehalose and forskolin was slightly higher than their theoretical additional effect (Figure 2C) and was still so after 12 h (Figure 2D). Interestingly, the additional effect of trehalose on the amplitude of the later maximum was already present at 15 mM trehalose, a dose not significantly elevating CRE-luc activity by itself (Figure 1A).

**Figure 3:**
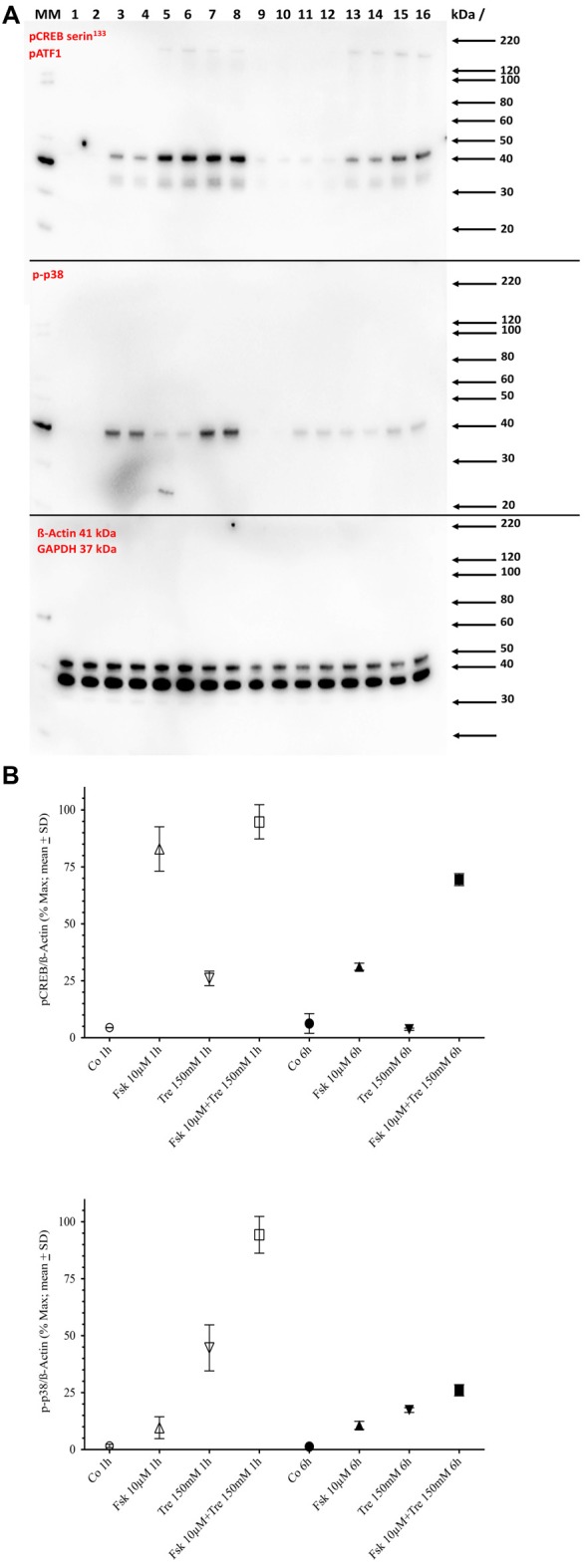
Panel **(A)** shows the Western blot images for serine 133 phosphorylated cAMP regulated element binding protein (pCREB; upper panel) and pp38 (middle panel) and ß-Actin plus GAPDH (lower panel) in untreated control cells (Co), cells treated with 10 μM forskolin (Fsk 10 μM), 150 mM trehalose (Tre 150 mM) or the combination of 10 μM forskolin and 150 mM trehalose (Fsk+Tre), extracted after 1 h and 6 h of two of the four replicates. Panel **(B)** shows the (semi)quantification analysis. After 1 h both trehalose (*p* < 0.01) and forskolin (*p* < 0.0001) elevate pCREB levels significantly. However, the combination of both is not significantly different. After 6 h trehalose treated cells are not significantly changed compared to control. Forskolin still elevates pCREB levels significantly (*p* < 0.0001) and the combination of both is significantly higher than forskolin alone (*p* < 0.001). After 1-h trehalose (*p* < 0.0001) elevated pp38 levels significantly, whereas forskolin had no effect. The combination of both is not was significantly higher than trehalose alone (*p* < 0.0001). After 6 h trehalose, forskolin and the combination of both were significantly elevated compared to control. However, there was no significant difference between trehalose, forskolin and the combination.

A conjecture so far not refuted is that elevation of CRE-dependent transcriptional activity needs at least transient phosphorylation of the CRE-binding protein CREB at the serine 133 site. Western blot analysis shows that both forskolin and trehalose application elevate the pCREB signal after 1-h application (Figure 3) with forskolin inducing a stronger signal than trehalose and the combination of both not different from forskolin alone. However, at 6 h of application, the effect of trehalose on pCREB is no longer significantly different from control levels, whereas forskolin is still elevated although not as much as after 1 h (Figure 3). Interestingly, the combination of forskolin and trehalose at 6 h is higher than both forskolin or trehalose and the theoretical sum of their intensities. This suggests separate pathways leading to the CRE-luc elevation by forskolin (which has been proposed to act via adenylate cyclase activity induction, cAMP elevation and PKA activation) and trehalose.

Since trehalose only transiently induced pCREB (elevated significantly after 1 h, but not after 6 h of application), we also tested the levels of the phosphorylated form of p38 (pp38), a protein kinase known to be activated by different agents and stimuli including stress conditions like hyperosmolar concentrations of substances. One-hundred and fifty micromolar trehalose indeed elevated pp38 levels significantly after 1 and 6 h of application (Figure 3; Supplementary Figure S1). Forskolin (10 μM) also elevated pp38 levels, but the combination of trehalose and forskolin was not significantly higher than trehalose or forskolin alone (Figure 3). Taken together the Western blot data for pCREB and pp38 do not support an important role for these two factors for the synergistic effects (higher and longer elevated amplitude) of the combination of trehalose and forskolin in the CRE-luc reporter gene activity.

**Figure 4:**
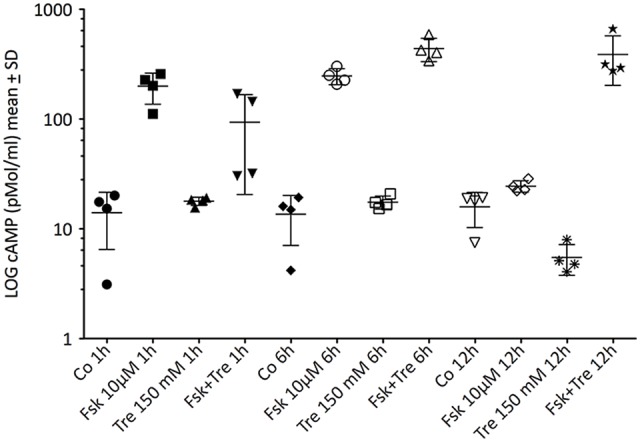
This figure shows the intracellular amount of cAMP in untreated control cells (Co), cells treated with 10 μM forskolin (Fsk 10 μM), 150 mM trehalose (Tre 150 mM) or the combination of 10 μM forskolin and 150 mM trehalose (Fsk+Tre), extracted after 1 h, 6 h and 12 h. Shown are the means with SD in pMol/ml, significances were calculated using ANOVA with Bonferroni correction as implemented in GraphPad Prism. After 1 h of treatment only Fsk 10 μM was significantly different from control (*p* < 0.05) trehalose and forskolin+trehalose did not significantly differ. After 6 h forskolin (*p* < 0.01) and forskolin+trehalose (*p* < 0.0001) were elevated over control. Moreover, forskolin+trehalose was significantly higher than forskolin (*p* < 0.05). After 12 h forskolin+trehalose was elevated compared to control (*p* < 0.0001) and was also significantly higher than forskolin (*p* < 0.0001).

Next, we investigated the concentration of intracellular cAMP under treatment with forskolin, trehalose and the combination of both in comparison to an untreated control after 1 h, 6 h and 12 h (Figure 4). As expected forskolin significantly elevated cAMP levels after 1- and 6-h treatment, while vehicle (control) and trehalose-treatment did not differ. The combination of forskolin and trehalose, however, was elevated also after 12 h of application whereas forskolin treatment alone was as low as untreated and trehalose-treated cells (Figure 4, right side). Trehalose alone did not change intracellular cAMP levels above or below control levels at any time point measured (Figure 4). Interestingly, trehalose in combination with forskolin does elevate cAMP levels over those induced by forskolin after 6 h and more so after 12 h (Figure 4). The prolonged elevation of forskolin-induced intracellular cAMP levels after 6 and 12 h may explain the further elevated and sustained CRE-luc activity observed (Figure 1B). However, the effect of trehalose on intracellular cAMP levels after 1 h of treatment does not support a role for cAMP in the trehalose-mediated inhibition after 1 h in the CRE-luc reporter gene activity (Figures 2A,B).

**Figure 5:**
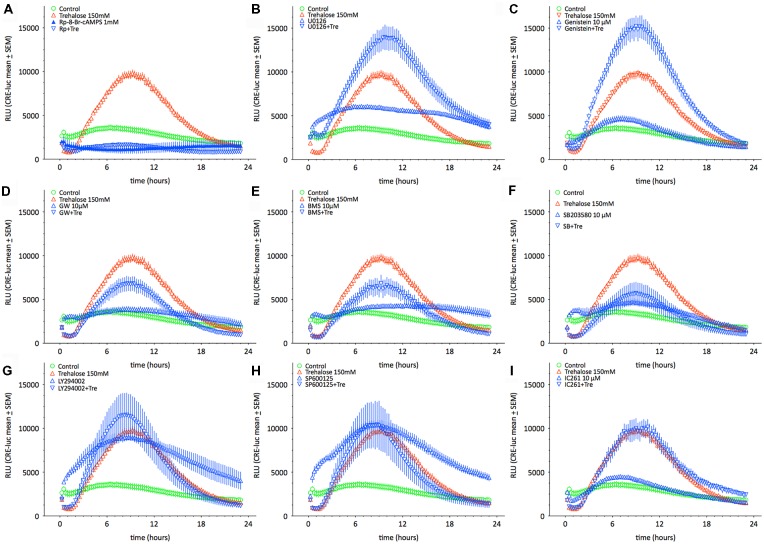
This figure shows the time-resolved influence of different protein kinase inhibitors on trehalose-elevated CRE-luc activity. Controls are represented by empty green circles (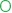), trehalose by empty red upward triangles (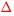), inhibitor alone by blue upward triangles (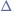) and the combination of trehalose with inhibitor by blue empty downward pointing triangles (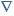). The inhibitors are **(A)** Rp-8-Br-cAMPS (1 mM), a cAMP-dependent protein kinase (PKA) antagonist (inhibited: *p* < 0.0001 vs. trehalose), **(B)** U0126 elevated: *p* < 0.05 vs. trehalose), an inhibitor of MEK1, the kinase that phosphorylates and activates p42/44 mitogen-activated protein kinase (p42/44MAPK) also known as Extracellular signal Regulated Kinase (“ERK”), **(C)** genistein (elevated: *p* < 0.01 vs. trehalose), an inhibitor for protein tyrosine kinases (PTK), three different inhibitors for p38, **(D)** GW), **(E)** BMS582949BMS, and **(F)** SB203580 (inhibited: *p* < 0.05 vs. trehalose), **(G)** the phosphatidyl-inositol-3-kinase (PI3K) inhibitor LY294002, **(H)** a “Jun N terminal kinase” (JNK) inhibitor SP60012 and **(I)** the casein kinase 1 delta and epsilon (CKId/e) inhibitor IC261. Inhibitor concentration was 10 μM if not indicated else. Shown are the mean RLU ± SEM of *N* = 4. ANOVA with Bonferroni post-test. Significances are trehalose vs. trehalose plus inhibitor.

**Figure 6:**
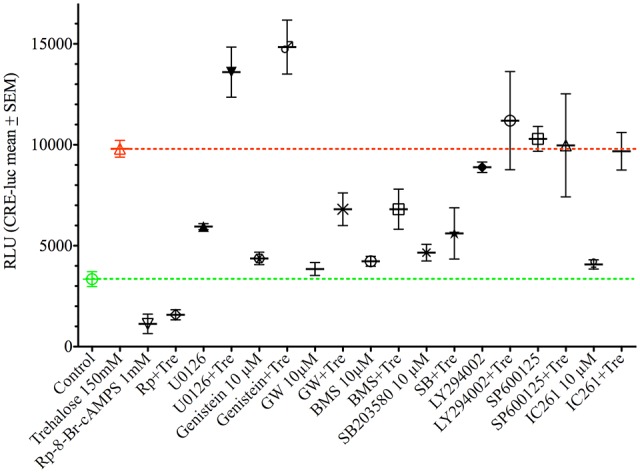
This figure shows the effect of different protein kinase inhibitors alone and in combination with trehalose-elevated as CRE-luc activity in RLU from Figure 5 at the amplitude maximum (9.25 h) for better comparison between the different agents. Shown are the mean RLU ± SEM of *N* = 4. ANOVA with Bonferroni post-test. Significances are as described in the legend of Figure 5.

As shown above changes in intracellular cAMP and pCREB levels cannot explain the elevation of CRE-luc activity by application of trehalose. However, pp38 was elevated after both 1 and 6 h of trehalose application (Figure 3; Supplementary Figures S1,S7,S8). To clarify the role of p38-dependent and other candidate signal transduction pathways trehalose was applied in combination with different protein kinase inhibitors in the next series of CRE-luc experiments (Figure 5). We tested Rp-8-Br-cAMPS, a PKA antagonist (Figure 5A), U0126, an inhibitor of MEK1, the kinase that phosphorylates and activates p42/44 mitogen-activated protein kinase (p42/44MAPK) also known as Extracellular signal Regulated Kinase (“ERK”; Figure 5B), genistein, an inhibitor for protein tyrosine kinases (PTKs; Figure 5C), three different inhibitors for p38, GW856553X (GW; Figure 5D), BMS582949 (BMS; Figure 5E) and SB203580 (Figure 5F), the phosphatidyl-inositol-3-kinase (PI3K) inhibitor LY294002 (Figure 5G), a “Jun N terminal kinase” (JNK) inhibitor SP600125 (Figure 5H) and the casein kinase 1 delta and epsilon (CKId/e) inhibitor IC261 (Figure 5I). A comparison of all stimulators and their inhibitors at the trehalose maximum time (9.25 h) is shown in Figure 6. Surprisingly, the only protein kinase inhibitor that abolished CRE-luc activity induced by trehalose was Rp-8-Br-cAMPS, a highly selective PKA inhibitor (Figure 5A). The effects of the other PK inhibitors were less profound. U0126, the inhibitor of MEK1 and genistein, the PTK inhibitor, both led to a steeper onset of the CRE-luc activity and a higher amplitude at the maximum (Figures 5B,C). The p38 inhibitors GW856553X, BMS582949 and SB203580 slowed the onset of CRE-luc activity and partially inhibited the trehalose effect (Figures 5D–F), whereas LY294002, SP600125 and IC261 had no effect (Figures 5G–I). Note that sole application of some inhibitors (U0126, LY294002 and SP600125) already elevated CRE-luc activity indicating a tonic suppressive effect of the kinases MEK1, PI3K and JNK on CRE-luc activity in HT22CRE cells (Figures 5B,G,H).

**Figure 7:**
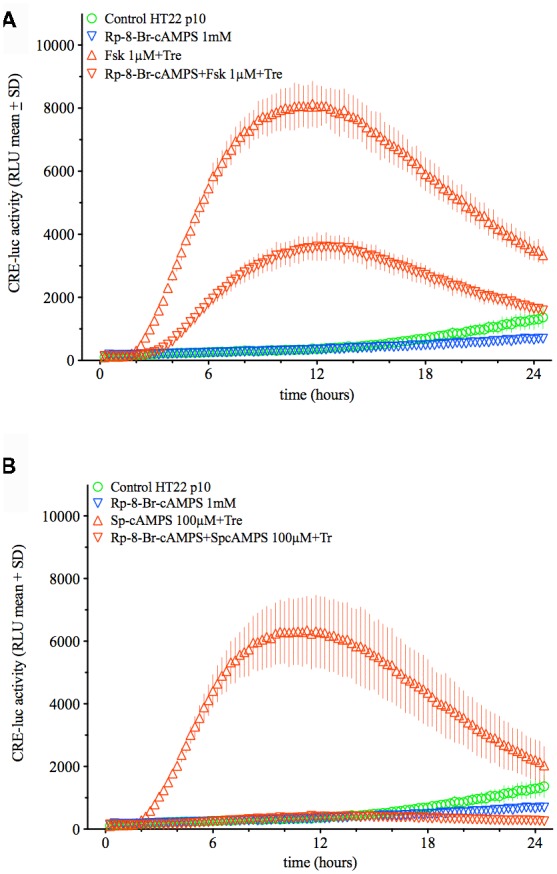
This figure shows the effect of the PKA antagonist Rp-8-Br-cAMPS (1 mM) on the combination of trehalose (150 mM) with forskolin (1 μM; upper graph) or Sp-cAMPS (100 μM; lower graph). Whereas the effect of forskolin plus trehalose is inhibited by about 60% (*p* < 0.0001; Fsk+Tre vs. Fsk+Tre+Rp-8-Br-cAMPS) the effect of Sp-cAMPS plus trehalose is inhibited by 100% (*p* < 0.0001; Sp-cAMPS+Tre vs. Sp-cAMPS+Tre+Rp-8-Br-cAMPS). Shown are the mean RLU + SD of *N* = 4. ANOVA with Bonferroni post-test.

Since PKA appeared as the central factor in trehalose-elevated CRE-luc activity, the effect of Rp-8-Br-cAMPS on the combination of trehalose with forskolin and Sp-cAMPS was tested next. While the effect of forskolin plus trehalose was significantly inhibited by the PKA antagonist Rp-8-Br-cAMPS (1 mM) but by only about 60% (*p* < 0.0001; Fsk+Tre vs. Fsk+Tre+Rp-8-Br-cAMPS) the effect of Sp-cAMPS plus trehalose was abolished (*p* < 0.0001; Sp-cAMPS+Tre vs. Sp-cAMPS+Tre+Rp-8-Br-cAMPS; Figure 7). These data point to a crucial role of PKA in both the effect of trehalose on CRE-luc activity in HT22 cells and the combined effect of trehalose with cAMP-elevating agents like Sp-cAMPS, forskolin and IBMX (data not shown). The finding that the effect of Sp-cAMPS which has no other target than PKA is abolished by Rp-8-Br-cAMPS, whereas the effect of forskolin is not fully inhibited points to additional actions of forskolin.

## Discussion

Data presented here show that trehalose application to HT22CRE cells leads to: (1) an induction of CRE-luc activity without elevation of cAMP levels; (2) a transient induction of CREB serine 133 phosphorylation; (3) an induction of phospho-p38; (4) a bi-phasic interaction with cAMP-elevating agents where trehalose is inhibitory to the forskolin-effect in the early phase (1–3 h) and synergistically activating the forskolin-effect in the second phase (6–12 h); and (5) a persisting elevation of intracellular cAMP levels and CRE-luc activity if combined with forskolin (or other cAMP elevating or PKA-activating agents). Furthermore, the effect of trehalose application on CRE-luc activity depends on PKA and to a lesser extent on p38 and is under tonic suppression by MEK1/p42/44MAPK and PTK activity. Finally, the combined effect of trehalose with forskolin and Sp-cAMPS also strongly depends on PKA.

There are principally three mechanisms how trehalose application can exert these effects:

1. The gluco-sensing mechanism established for pancreatic beta-cells. For this mechanism trehalose would have to be converted to glucose. This would demand the presence of the trehalose converting enzyme, trehalase. There is so far no evidence for the presence of trehalase in mammalian neuronal cells (but in astrocytes; Martano et al., 2017). Furthermore, own attempts to identify trehalase in HT22 cells failed (data not shown).
2. The second mechanism is signaling via the sweet taste receptors, Tas1/2/3R. These receptors bind sugars like glucose, sucrose or mannitol and activate, via G-protein-coupling, intracellular second messenger pathways, in taste buds in the tongue mostly the G-protein Gs thereby elevating intracellular cAMP. Interestingly, Tas1R3 knockout mice show profound nervous system failures including altered behavior, memory and social behavior (Martin et al., 2017). Our data clearly show no elevation (or reduction) of intracellular cAMP levels after trehalose application.
3. The third mechanism depends on changes in the extracellular osmolarity. For this mechanism the high level of trehalose in the extracellular medium would lead to water loss of the cells and the shrinking of the cells activates mechanic stress in the plasma membrane. This would imply that other sugars also induce CRE-luc activity and indeed there is preliminary evidence with glucose and mannitol application which induce similar effects (Supplementary Figure S10).

The signal transduction mechanisms of trehalose may include the stress kinase p38, but this kinase does not appear to be the main factor since of the three different inhibitors tested for p38 only S203580 partly inhibited trehalose-induced CRE-luc activity. Other kinases (p42/44MAPK and PTKs) appeared to act inhibitory on the trehalose effect, while PI3K and casein kinase 1delta/epsilon had no influence.

How can application of trehalose lead to prolonged elevation of cAMP, persistently elevated pCREB levels and synergistically elevated and sustained CRE-luc activity? Since trehalose itself does not elevate cAMP levels it may stabilize adenylate cyclase activity by changing cell membrane structure thereby stabilizing adenylate cyclase catalysis. Trehalose may also induce processes that lead to elevated and/or sustained ATP supply for adenylate cyclase. At this point however, this is only speculation. Another mechanism may be induction of persistent PKA activation. This has been proposed for other biological systems like the brain of the fruit fly (Buxbaum and Dudai, 1989). In human endometrial stromal cells treatment with the hormone Relaxin has been shown to induce a prolonged elevation of cAMP with a subsequent disappearance of the PKA regulatory subunit RIα (Telgmann et al., 1997). In the latter two systems sustained elevation of cAMP rendered most of the present PKA dissociated into its regulatory (R) and catalytic (C) subunits. Under such conditions regulatory subunits are prone to proteolytic degradation (presumably via ubiquitinylation and subsequent proteasomal degradation) and the catalytic subunits stay active and can keep phosphorylating target proteins. In the human endometrium this has been shown to lead to the complete disappearance of RIα, but RIIα and RIIβ were not affected. Preliminary analysis of the different PKA isoforms in HT22 cells showed predominantly the presence of RIIα and RIIβ and very small amounts of RIα and RIβ (data not shown). Another effect may be prolongation of the activity of the PKA catalytic activity by phosphorylation. Indeed, one such phosphorylation by PTKs has been described (Caldwell et al., 2011). However, if the phosphorylation of the PKA catalytic subunit is important for the effects of trehalose observed here remains to be determined. Of cause many other signaling systems may have been activated by the application of trehalose and were overseen. It remains to be determined how exactly the trehalose effect on CRE-luc activity can be explained.

## Data Availability

The datasets generated and analyzed for this study are available from the author upon request.

## Author Contributions

EM did all experiments, analyzed all data and wrote the manuscript.

## Conflict of Interest Statement

The author declares that the research was conducted in the absence of any commercial or financial relationships that could be construed as a potential conflict of interest. The reviewer OR declared a past co-authorship with the author to the handling editor.
