# Supplementary material for: Trehalose Activates CRE-Dependent Transcriptional Signaling in HT22 Mouse Hippocampal Neuronal Cells: A Central Role for PKA Without cAMP Elevation
Source: Front Mol Neurosci. 2018 Oct 22;11:386. doi: 10.3389/fnmol.2018.00386 (PMC6204353; doi:10.3389/fnmol.2018.00386)
Supplement: Supplementary file 1 [file Data_Sheet_1.pdf]

Supplementary Material

Trehalose activates CRE-dependent transcriptional signalling in HT22 mouse hippocampal neuronal cells: a central role for PKA without cAMP elevation

Erik Maronde*

*** Correspondence:** Corresponding Author: e.maronde@em.uni-frankfurt.de

1 Supplementary Figures and Tables

1.1 Supplementary Figures

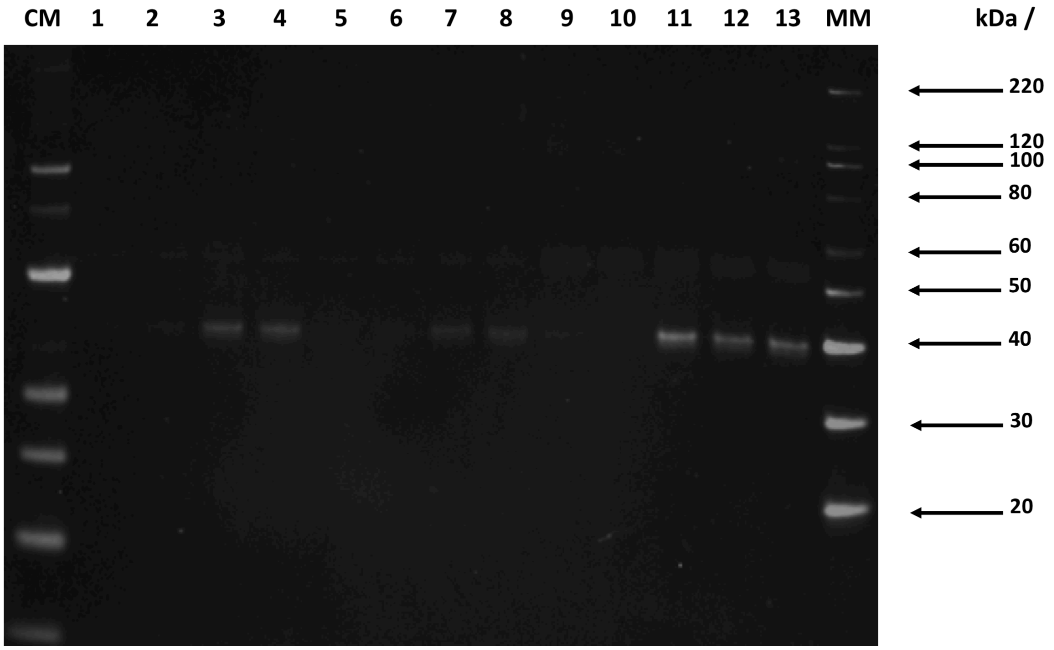

Supplementary Figure 1. Supplementary figure 1 shows a Western blot detecting pp38 with extracts from HT22 wildtype (wt; 1-4), HT22CRE (5-8) and primary hippocampal neuronal cultures (9-13) from C3H/HeN mice. Lanes 1,2,5,6 and 9,10 show untreated control cells, lanes 3,4,7,8 and 11-13 were treated with 150 mM trehalose for one hour.

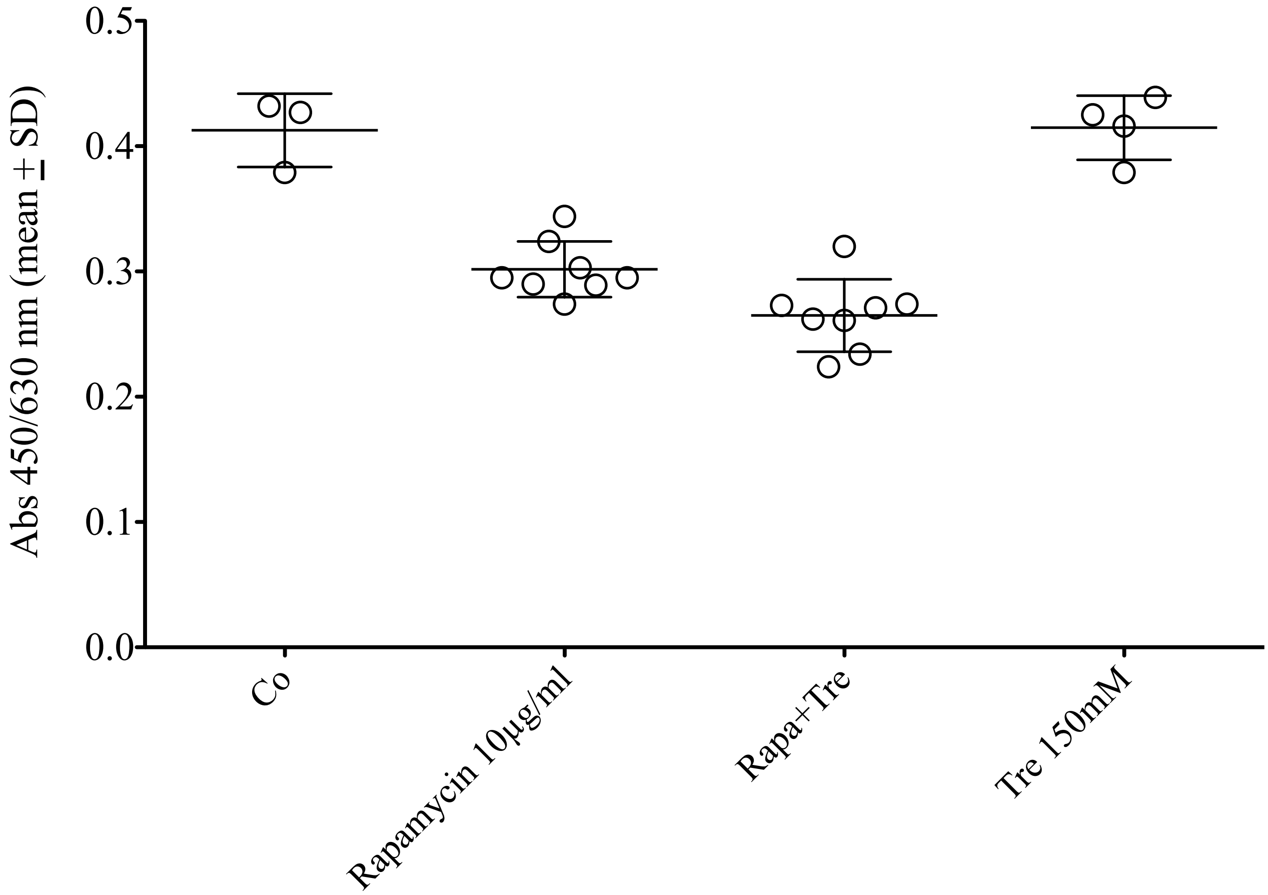

Supplementary Figure 2. Supplementary figure 2 shows a WST-1 assay with HT22CRE cells treated with 10µg/ml Rapamycin, 150mM trehalose or both after 24 hours. Note that the WST-1 activity in the trehalose treated cells does not differ from control whereas Rapamycin treatment alone or in combination with trehalose reduced WST-1 activity significantly against both control and trehalose treated cells. Co versus rapamycin $p<0.0001$, trehalose versus rapamycin $p<0.0001$, rapamycin versus trehalose+rapamycin (n.s.). Means $\pm$ standard deviation (SD), N=3-8. ANOVA with Bonferroni post-test.

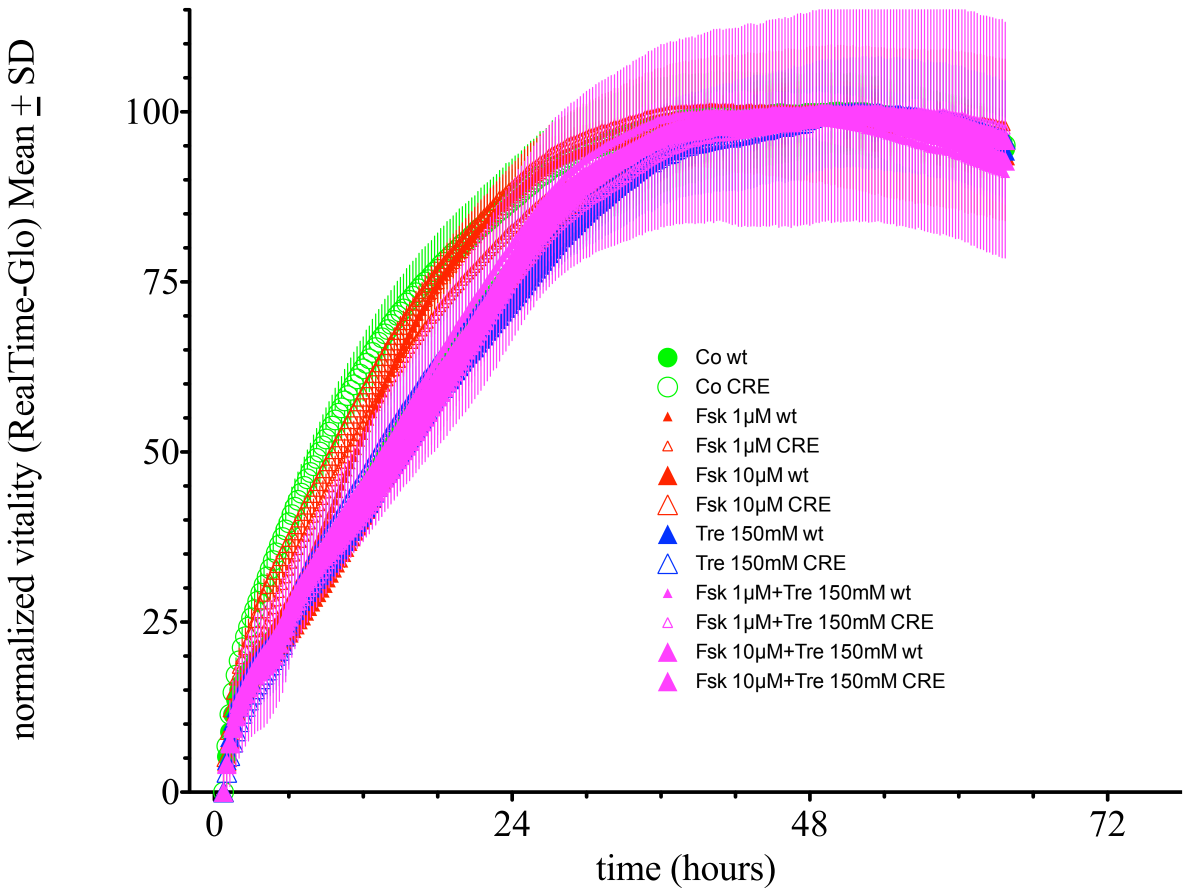

Supplementary Figure 3. Supplementary figure 3 shows a RealTime Glo assay with HT22 wt and HT22CRE cells treated with 150mM trehalose alone or in combination with 1µM or 10µM forskolin measured every 15 minutes over a total time of 60 hours. Note that the RealTime Glo activity does not differ between the different combinations of agents indicating no significant influence of the treatments on the vitality of the cells in the investigated time frame. Shown are the means ± standard deviation (SD) of N=4. ANOVA with Bonferroni post-test.

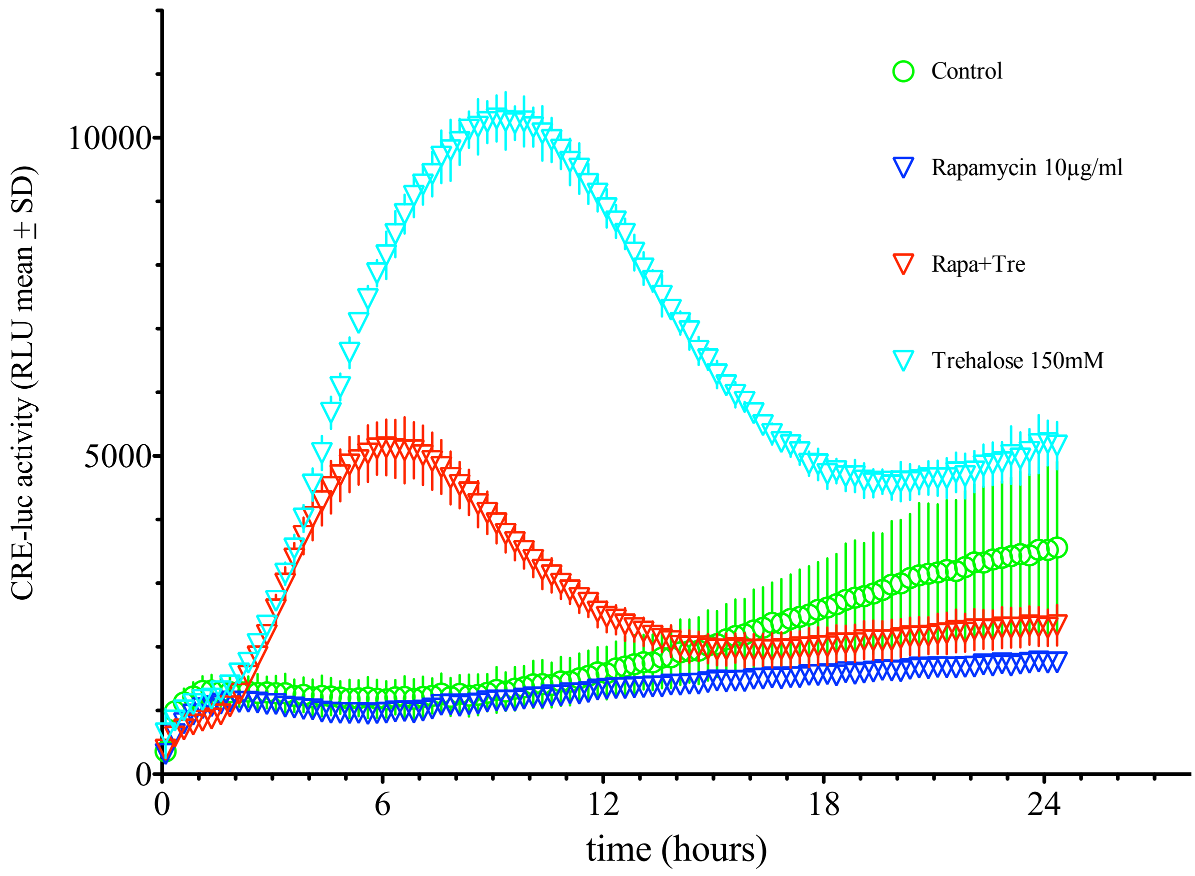

Supplementary Figure 4. Supplementary figure 4 shows that the “autophagy inducers” rapamycin (10µg/ml) and trehalose (150mM) display very different influence on HT22CRE luciferase activity. Whereas trehalose induced HT22CRE luciferase activity, rapamycin alone had no influence, but in combination inhibited trehalose-induced activity. Shown are the means ± standard deviation (SD), N=4. ANOVA with Bonferroni post-test.

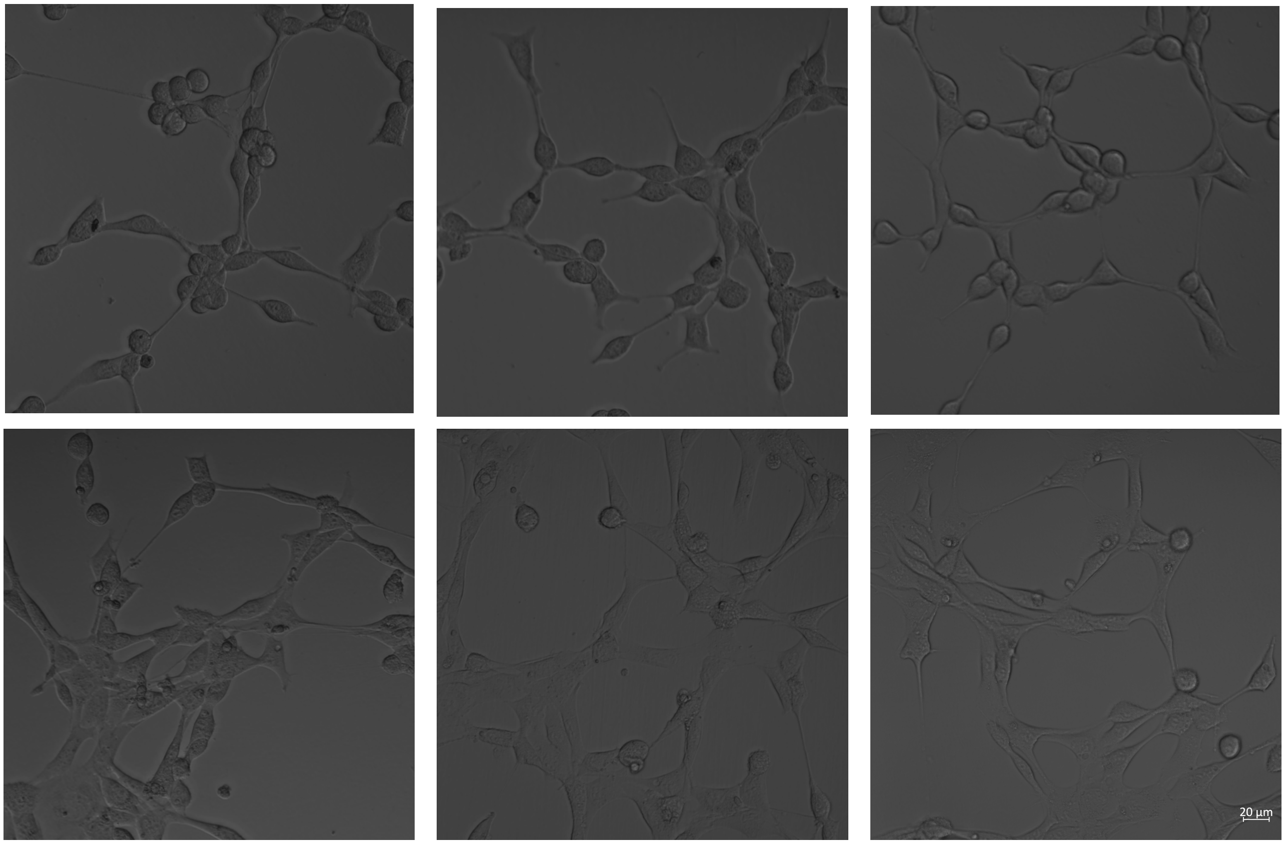

Supplementary Figure 5. Supplementary figure 5 shows representative images of living HT22 wt (upper panel) and HT22CRE cells (lower panel) plated at the same density as in the reporter gene assay as the cells present themselves at the start of a reporter gene assay. Scale bar represents 20µm. Images were taken from transparent bottom 96 well plates using an inverted Zeiss LSM 710 confocal microscope. My thanks for taking the pictures go to Dr. Christoph Schürmann, Center of Physiology, Department of Medicine, Goethe University, Frankfurt, Germany.

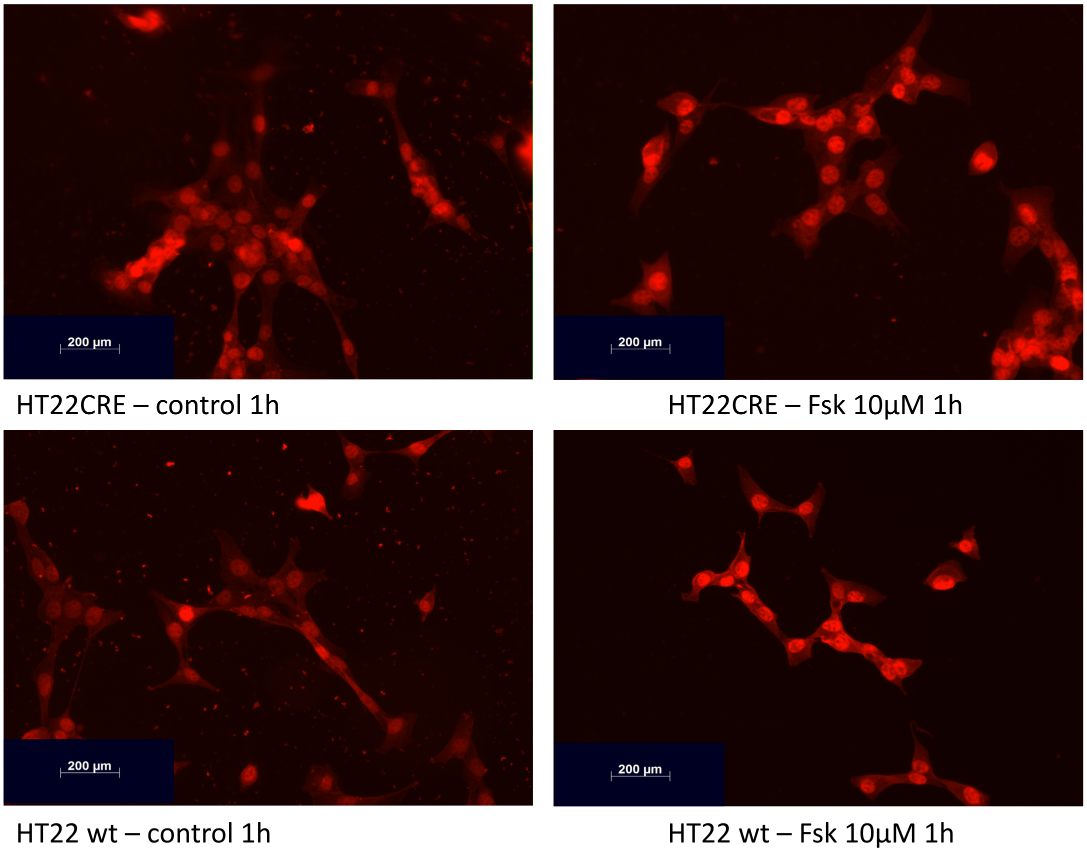

Supplementary Figure 6. Supplementary figure 6 shows representative images of living HT22CRE (upper panel) and HT22 wt cells (lower panel) plated at the same density as in the reporter gene assay, treated for one hour with forskolin (10µM) (right side) or left untreated (left side) and stained with a rabbit-anti-pCREB antibody (Cell Signalling 87G3, 1:1000) and Alexa568 secondary antibody. Note the brighter and more rounded nuclei under forskolin treatment (right side). My thanks for assistance with the immunocytochemistry to Dr. Sonja Meimann and for taking the pictures go to Prof. Dr. Dr. Abdelhaq Rami, Dr. Senckenbergische Anatomie, Institute for cellular and molecular Anatomy, Department of Medicine, Goethe University, Frankfurt, Germany.

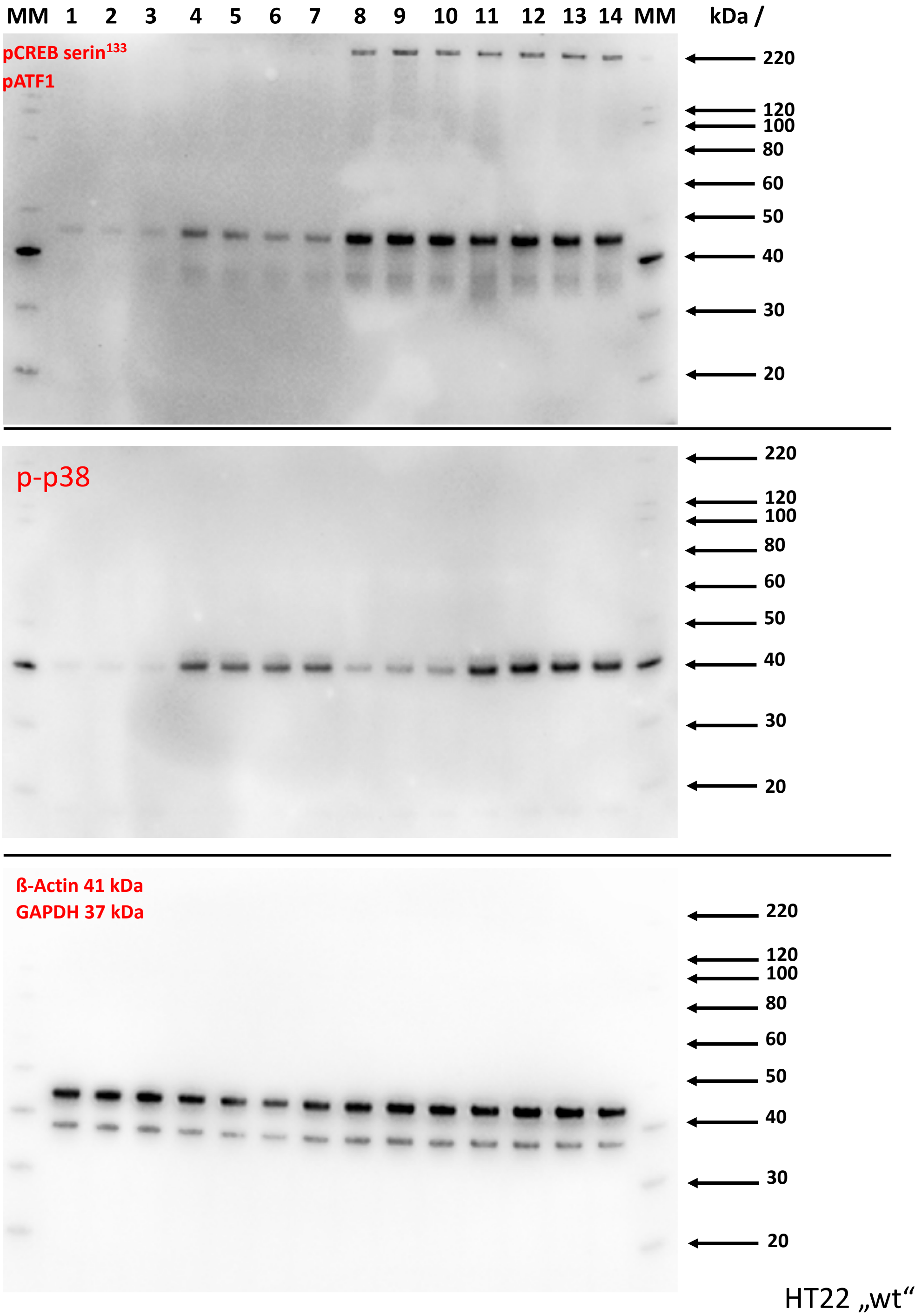

Supplementary Figure 7. Supplementary figure 7 shows Western blot images of untreated HT22wt (lanes 1-3), treated with 150mM trehalose (lanes 4-7), forskolin 10µM (lanes 8-10) or forskolin plus trehalose (11-14) for one hour and stained with a rabbit-anti-pCREB (CS #87G3, 1:1000; upper image), phospho p38 (pp38; 1:1000; middle image) or mouse anti-ß-actin (1:10.000) combined with mouse anti-GAPDH (1:2000) and HRP-conjugated secondary antibodies as described in Materials and Methods.

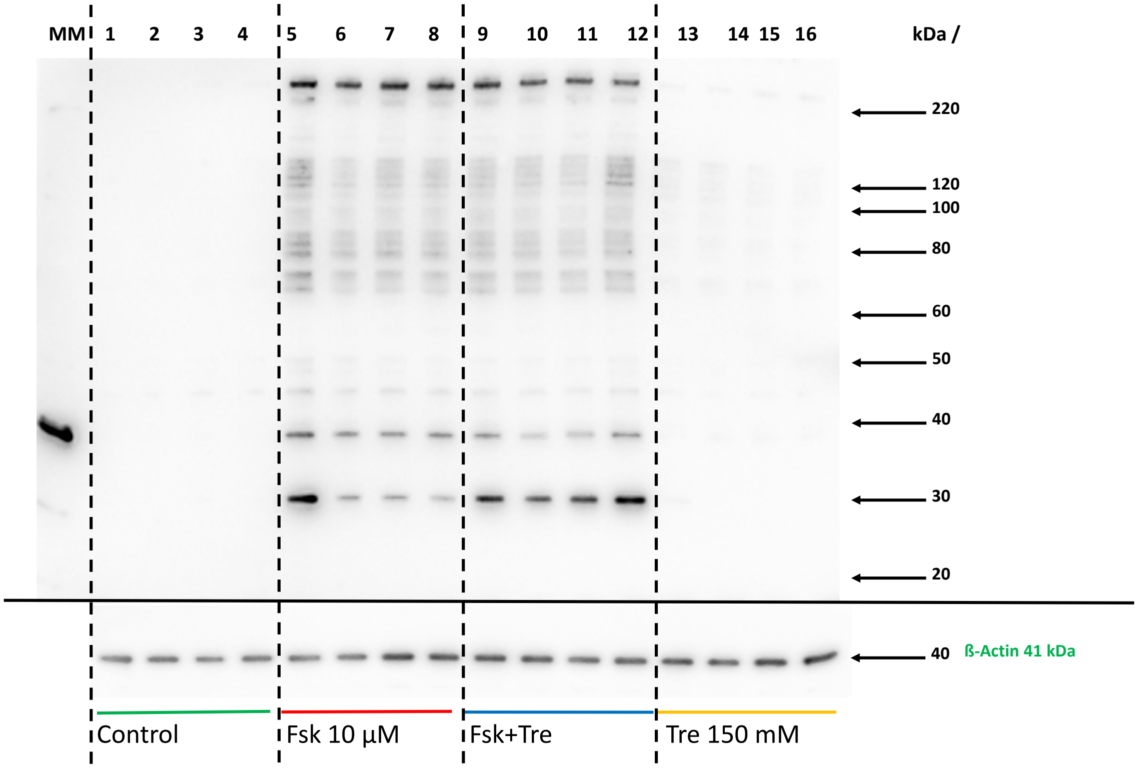

Supplementary Figure 8. Supplementary figure 8 shows a Western blot images of untreated HT22CRE (lanes 1-4), treated with 10µM forskolin (lanes 5-8), forskolin plus trehalose (9-12) or 150mM trehalose (lanes 13-16) for one hour and stained with a rabbit-anti-pPKAS (1:5000). Mouse anti-ß-actin (1:10.000) is shown as a loading control. Note the faint signals under trehalose treatment indicating a weak activation of an AGC kind of kinase.

Western blot (semi)quantification procedure

1. Chemiluminescence procedure (see Materials and Methods)

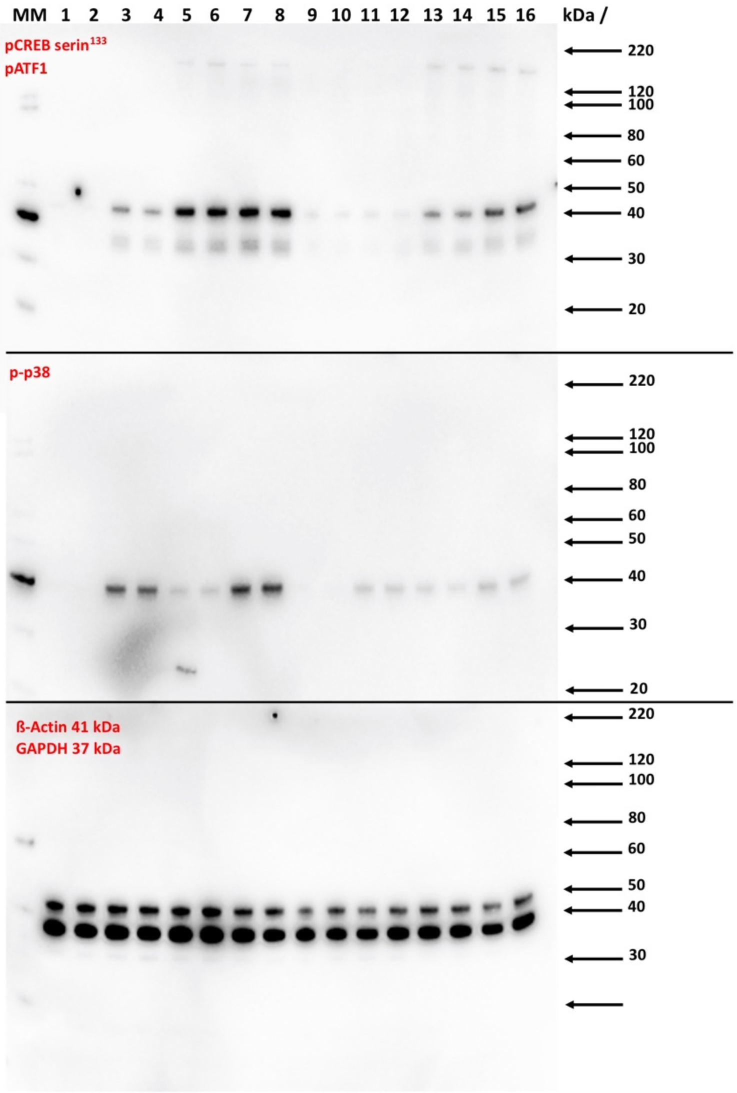

MM	= Magic Marker
1,2	= Control HT22CRE 1h
3,4	= Trehalose 150mM HT22CRE 1h
5,6	= Forskolin 10µM HT22CRE 1h
7,8	= Tre+Fsk HT22CRE 1h
9,10	= Control HT22CRE 6h
11,12	= Trehalose 150mM HT22CRE 6h
13,14	= Forskolin 10µM HT22CRE 6h
15,16	= Tre+Fsk HT22CRE 6h
kDa	= kilo Dalton

2. Select lanes and region to be measured on the *.tif-File using QuantiScan™:

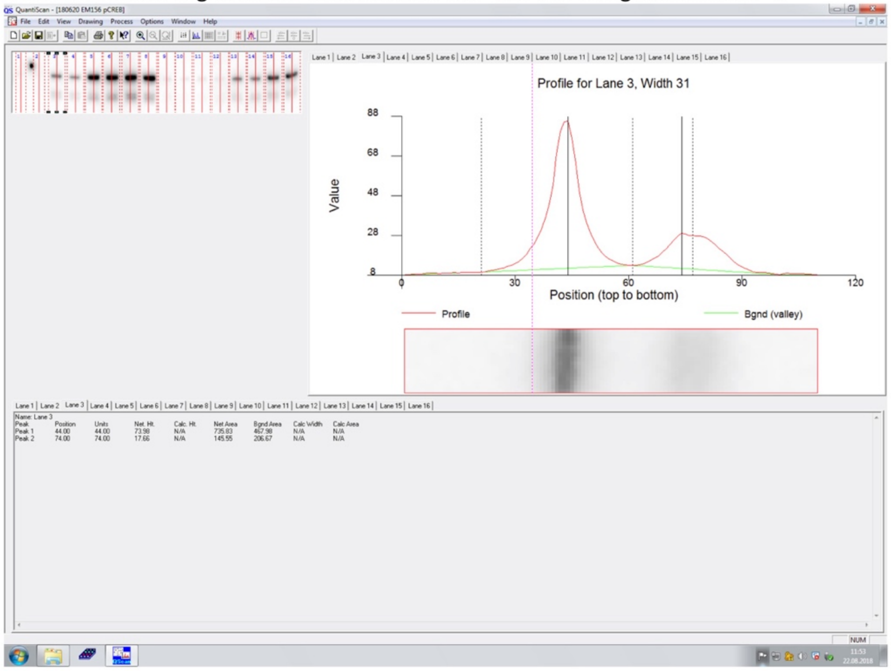

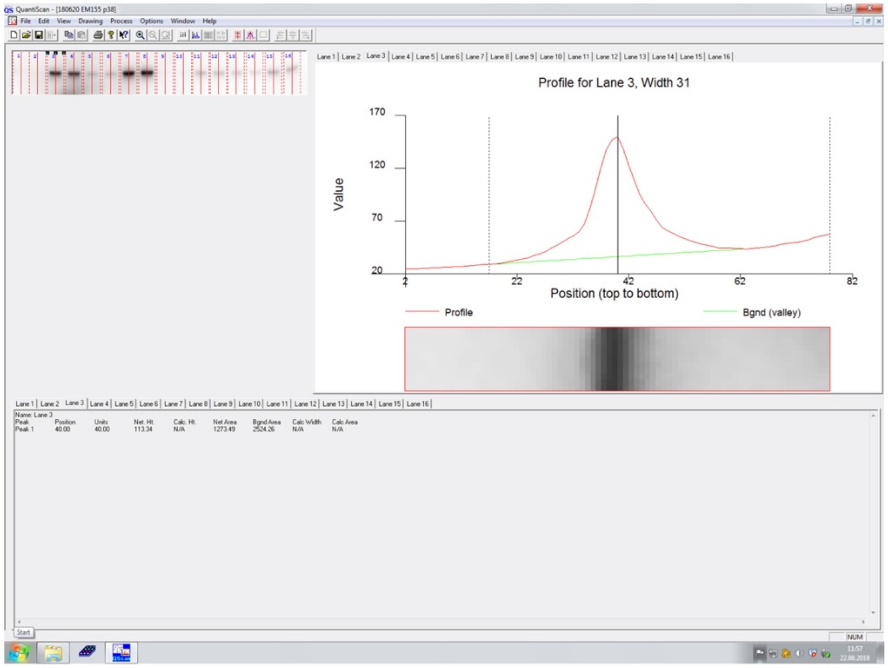

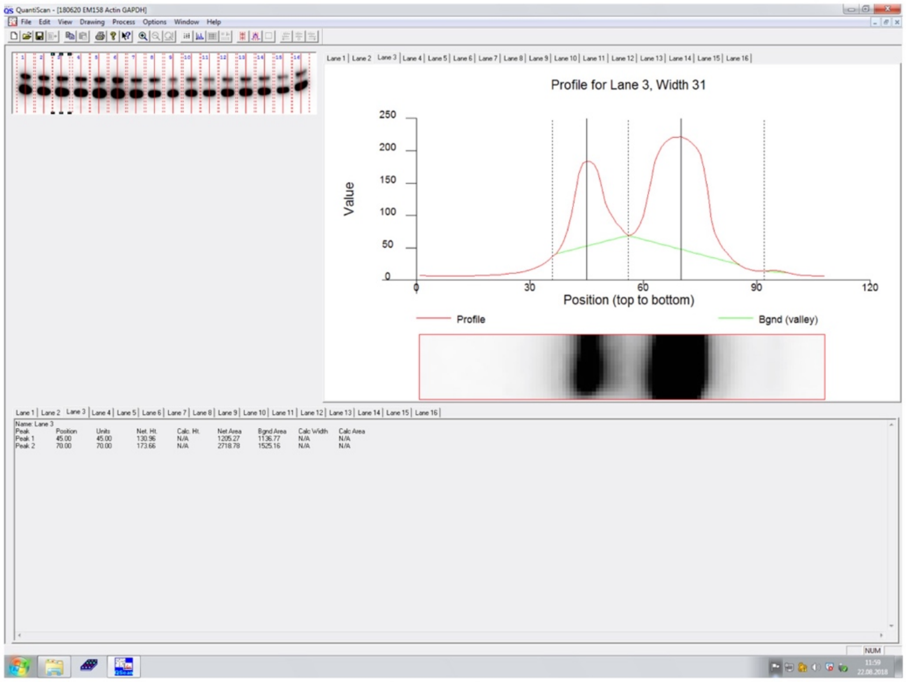

3. Divide the QuantiScan “area density” values for pCREB or p-p38 by those for ß-Actin or GAPDH
4. Put normalized (e.g. % of Max/corr by ß-Actin) “Area density” values into GrapPad Prism

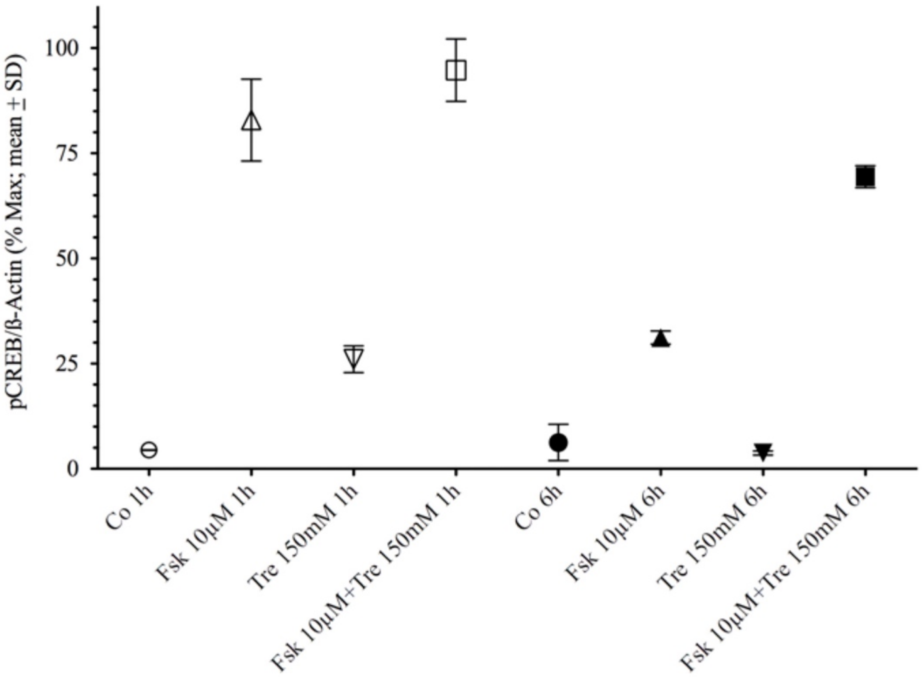
pCREB/ß-Actin (% Max; mean ± SD)
100
75
50
25
0
Co 1h
Fsk 10μM 1h
Tre 150mM 1h
Fsk 10μM+Tre 150mM 1h
Co 6h
Fsk 10μM 6h
Tre 150mM 6h
Fsk 10μM+Tre 150mM 6h

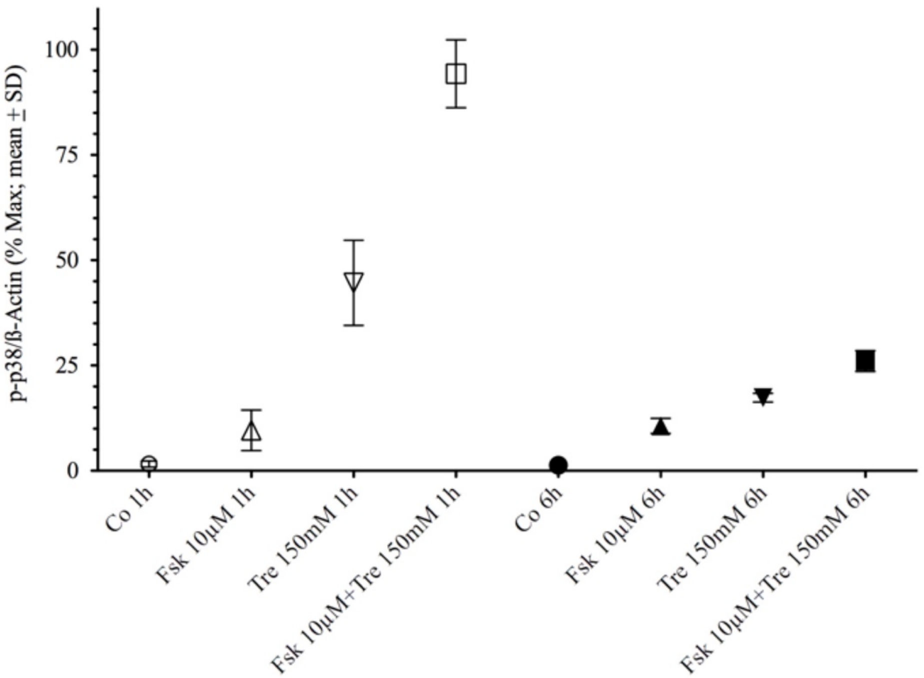
p-p38/ß-Actin (% Max; mean ± SD)
100
75
50
25
0
Co 1h
Fsk 10μM 1h
Tre 150mM 1h
Fsk 10μM+Tre 150mM 1h
Co 6h
Fsk 10μM 6h
Tre 150mM 6h
Fsk 10μM+Tre 150mM 6h

Bonferroni's Multiple Comparison Test	Mean Diff,	t	Significant? P < 0,05?	Summary
Co 1h vs Fsk 10µM 1h	-78,45	16,16	Yes	****
Co 1h vs Tre 150mM 1h	-21,60	4,449	Yes	*
Co 1h vs Fsk 10µM+Tre 150mM 1h	-90,30	18,60	Yes	****
Co 6h vs Fsk 10µM 6h	-24,95	5,139	Yes	**
Co 6h vs Tre 150mM 6h	2,500	0,5149	No	ns
Co 6h vs Fsk 10µM+Tre 150mM 6h	-63,20	13,02	Yes	****

pCREB/corrected by ß-Actin

Bonferroni's Multiple Comparison Test	Mean Diff,	t	Significant? P < 0,05?	Summary
Co 1h vs Fsk 10µM 1h	-8,000	1,595	No	ns
Co 1h vs Tre 150mM 1h	-43,05	8,584	Yes	***
Co 1h vs Fsk 10µM+Tre 150mM 1h	-92,70	18,48	Yes	****
Co 6h vs Fsk 10µM 6h	-9,350	1,864	No	ns
Co 6h vs Tre 150mM 6h	-16,05	3,200	No	ns
Co 6h vs Fsk 10µM+Tre 150mM 6h	-24,75	4,935	Yes	**

p-p38/corrected by ß-Actin

Supplementary Figure 9. Supplementary figure 9 shows the Western blot (semi)quantification procedure used in the present work.

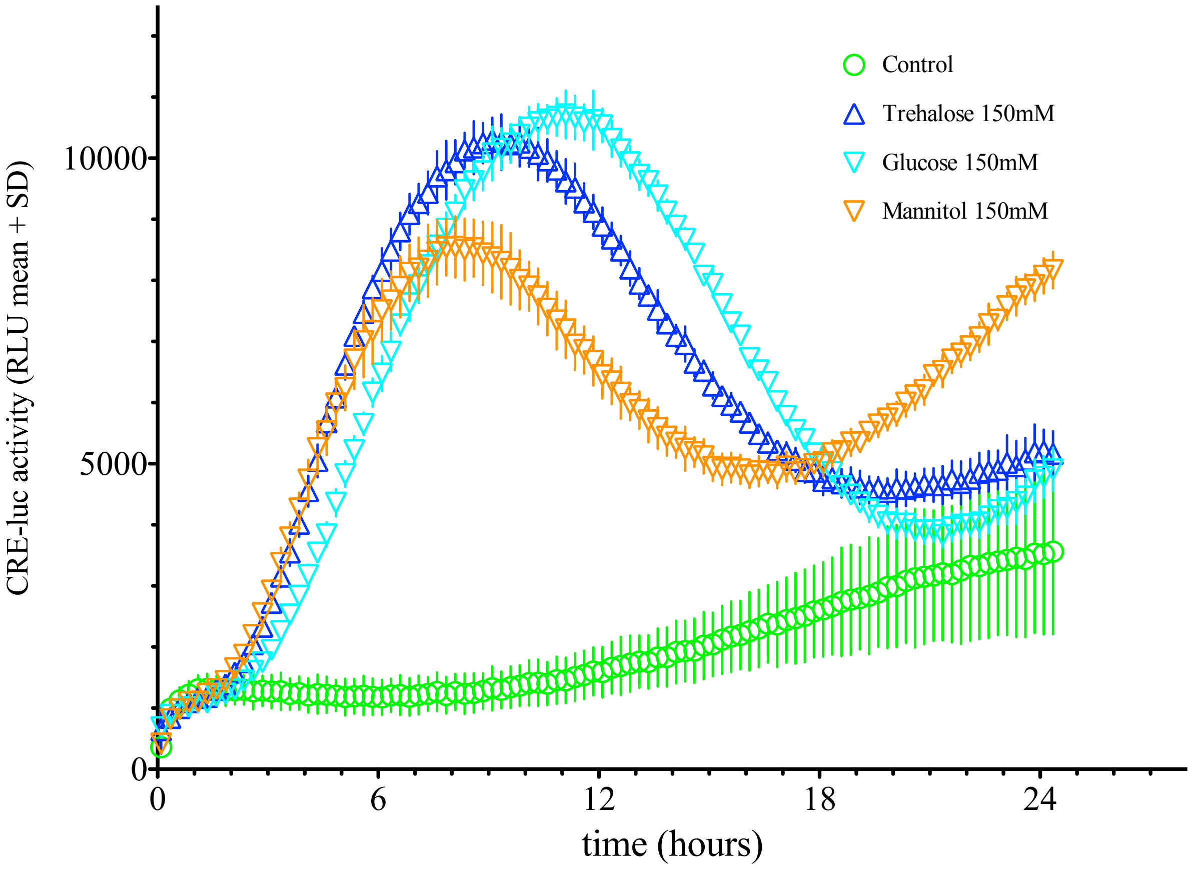

Supplementary Figure 10. Supplementary figure 10 shows that not only trehalose, but also glucose, a reducing and mannitol, a non-reducing, monosaccharide induce CRE-luciferase activity in HT22CRE cells. Shown are the means + standard deviation (SD), N=4. ANOVA with Bonferroni post-test.

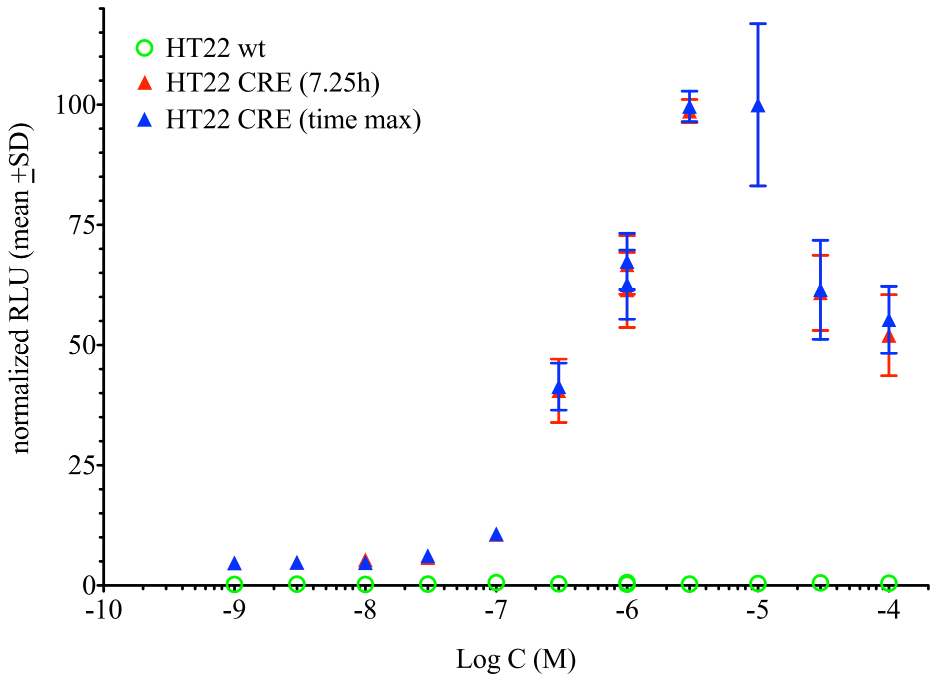

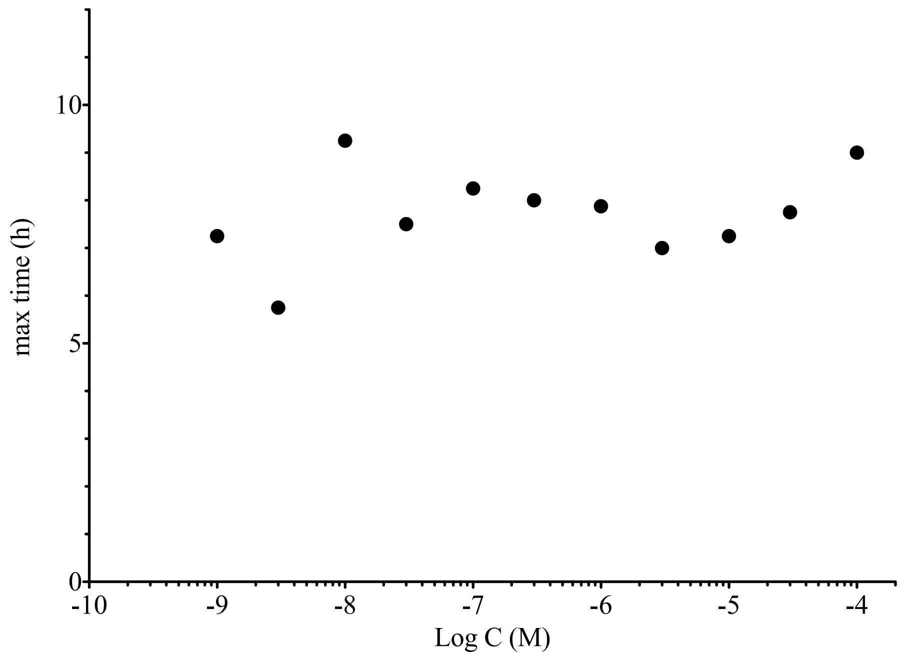

Supplementary Figure 11. Supplementary figure 11 shows that the maximum of CRE luciferase response changes with the dose of forskolin applied to HT22CRE cells. However, this does not significantly alter the dose response curve. Shown are the means ± standard deviation (SD), N=4. ANOVA with Bonferroni post-test.

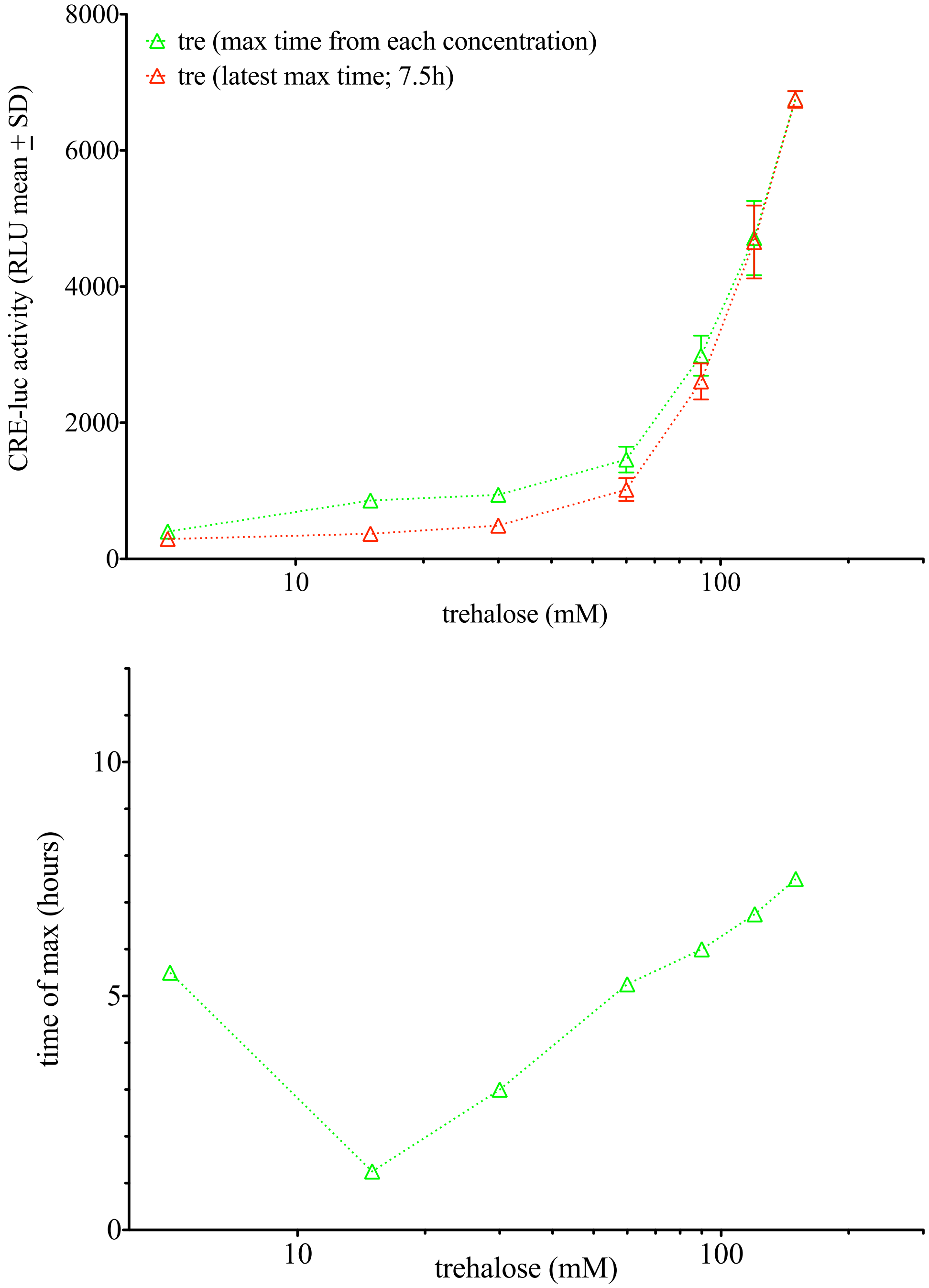

Supplementary Figure 12. Supplementary figure 12 shows replotted data from Figure 1 and shows that the maximum of CRE luciferase response changes with the dose of trehalose applied to HT22CRE cells. However, this does not significantly alter the dose-response curve. Shown are the means ± standard deviation (SD), N=4. ANOVA with Bonferroni post-test.
